# Inhibition of Mitochondrial ROS by MitoQ Alleviates White Matter Injury and Improves Outcomes after Intracerebral Haemorrhage in Mice

**DOI:** 10.1155/2020/8285065

**Published:** 2020-01-04

**Authors:** Weixiang Chen, Chao Guo, Zhengcai Jia, Jie Wang, Min Xia, Chengcheng Li, Mingxi Li, Yi Yin, Xiaoqin Tang, Tunan Chen, Rong Hu, Yujie Chen, Xin Liu, Hua Feng

**Affiliations:** Department of Neurosurgery, Southwest Hospital, Third Military Medical University (Army Medical University), 29 Gaotanyan Street, Shapingba District, Chongqing 400038, China

## Abstract

White matter injury (WMI) is an important cause of high disability after intracerebral haemorrhage (ICH). It is widely accepted that reactive oxygen species (ROS) contributes to WMI, but there is still no evidence-based treatment. Here, mitoquinone (MitoQ), a newly developed selective mitochondrial ROS scavenger, was used to test its neuroprotective potential. The data showed that MitoQ attenuated motor function deficits and motor-evoked potential (MEP) latency prolongation. Further research found that MitoQ blunted the loss of oligodendrocytes and oligodendrocyte precursor cells, therefore reduced demyelination and axon swelling after ICH. In the in vitro experiments, MitoQ, but not the nonselective antioxidant, almost completely attenuated the iron-induced membrane potential decrease and cell death. Mechanistically, MitoQ blocked the ATP deletion and mitochondrial ROS overproduction. The present study demonstrates that the selective mitochondrial ROS scavenger MitoQ may improve the efficacy of antioxidant treatment of ICH by white matter injury alleviation.

## 1. Introduction

The prevalence rate of intracerebral haemorrhage (ICH) is approximately 120/100000 [1]. Fifty-eight percent of ICH patients die within one year, and two-thirds of survivors remain moderately or even severely disabled [2, 3]. Serious secondary brain injury (SBI) is the major cause of the poor prognosis of the patient after ICH, which includes white matter injury, inflammation, and neuronal death [4]. Among these processes, white matter injury- (WMI-) induced motor function deficit is a serious complication affecting the quality of life of patients after ICH [5]. However, there is still no medicine available for WMI after intracerebral haemorrhage.

Reactive oxygen species (ROS) are the primary inducement of secondary injury after ICH [6]. Excessive accumulation of ROS can induce significant cell death and tissue damage [7]. Since the mitochondria are the main source of ROS, mitochondria enrichment and hyperoxia consumption in the central nervous system lead to the tissue being susceptible to oxidative stress injury [8, 9]. Oligodendrocyte is rich in lipids and is prone to oxidative stress damage, which leads to white matter injury [10]. Several antioxidants showed promising results but failed in the clinical trial of intracerebral haemorrhage [11, 12]. ROS are mainly produced by the Fenton reaction induced by iron overload after ICH, which occurs primarily in the mitochondria [13–15]. And the selective mitochondrial ROS scavengers are reported superior to nonselective ROS scavengers in the treatment of many redox diseases involving mitochondrial dysfunction [16–18]. Therefore, it is urgent to explore the protective effect of selective mitochondrial ROS scavenger on secondary injury of ICH.

Mitoquinone (MitoQ) is a selective mitochondrial antioxidant that accumulates in high concentrations in the mitochondria. The compound which passes easily through the blood-brain barrier rapidly accumulates in the brain [19, 20]. Although the administration of MitoQ can reduce mitochondrial oxidative damage in in vitro experiments such as erastin-mediated ferroptosis and in vivo experiments such as myocardial injury models [21, 22], it still needs to be investigated after induction after ICH. To explore the role of selective targeting mitochondrial ROS in white matter damage of ICH and its related mechanisms, MitoQ was administrated and demyelination, white matter injury, and neurological deficits were explored after ICH in this study.

## 2. Materials and Methods

### 2.1. Animal Model

All animal procedures were approved by the Animal Care and Use Committee of the National Institute on Aging Intramural Research Program. Seven-week-old C57BL/6N mice weighing 23–26 g were purchased from Army Medical University. The animals were randomly divided into different experimental groups.

The animals were anesthetized with halothane (70% N_2_O and 30% O_2_; 4% induction, 2% maintenance, China), immobilized on a stereotactic instrument (RWD Life Sciences Ltd. China), and injected with 25 *μ*l of autologous blood into the right caudate nucleus. The following coordinates were used, as described previously, from bregma: 0.8 mm anteriorly, 2.5 mm laterally, and 3.0 mm deep [23]. The craniotomy was finished with bone wax, and sutures were applied to the scalp. During the entire experiment and recovery, the body temperature of the animals was maintained at 37 ± 0.5°C. Sham-operated mice were subjected to needle insertion only.

MitoQ was purchased from BioVision (B1309, USA, dissolved in a 1 : 1 ratio of ethanol to water and dissolved in 1 mL 0.9% sterile NaCl at a final concentration of 1 mg/mL) and administered intraperitoneally (i.p.) 1 hour and 24 hours after ICH (4 mg/kg). The ICH+vehicle group received an equal volume of solvent at the corresponding time point as the ICH+MitoQ group [20, 24].

### 2.2. Immunohistochemistry

The brains were removed after perfusion with the fixative 4% paraformaldehyde and then immersed in 30% sucrose in phosphate-buffered saline (PBS). Serial sections were cut on a freezing microtome, blocked, and incubated in the following primary antibodies: goat anti-MBP (diluted 1 : 500, Santa Cruz, sc-13914, USA), rabbit anti-Neurofilament 200 (NF200) (1 : 200; Sigma-Aldrich, N4142, USA), mouse anti-APC (or Ab-7, CC-1) (1 : 500; Merck Millipore, OP80, USA), and rat anti-NG-2 (NG2 chondroitin sulfate proteoglycan; 1 : 200; Millipore; AB5320, USA). After washing, the sections were incubated with the appropriate fluorescent secondary Alexa Fluor 488- or Alexa Fluor 555-conjugated antibody (diluted 1 : 1000, Invitrogen, USA) and counterstained with DAPI. Images of the perihematomal region in each section were captured by a Zeiss microscope (LSM780; Zeiss, Germany). Randomly selected microscopic fields on each of the three consecutive sections from each brain were analyzed by a blinded investigator.

### 2.3. Transmission Electron Microscopy

For the transmission electron microscopy (TEM), the animals were perfused with 1.25% glutaraldehyde and 2% paraformaldehyde in 0.1 M PB after an initial flush with isotonic saline. Then, the brains were rapidly removed and fixed for at least three days at 4°C. The tissues were rinsed and post fixed with 1% OsO_4_ in PB for two hours, counterstained with uranyl acetate, dehydrated in a graded series of acetone, infiltrated with propylene oxide, and embedded in Epon. Ultrathin sections (~60 nm) were cut by an ultramicrotome (LKB-V, LKB Produkter AB, Bromma, Germany) and observed under a transmission electron microscope (Tecnai 10; Philips, Netherlands) [25]. Random images of 12 different fields of view were selected for each animal for the statistical analysis of myelinated axons. The *g*-ratios of the myelinated fibers were calculated as the ratio of the diameter of the axon to the diameter of the axon and myelin sheath using ImageJ software (ImageJ 1.8; NIH, Bethesda, MD, USA), and at least 60 myelinated fibers from each animal were analyzed [26].

### 2.4. Behavioral Tests

#### 2.4.1. Beam Walking Test

The mouse was allowed to cross a round wooden beam with a diameter of 1.5 cm and a length of 70 cm. The mice were required to completely cross the beam to obtain a corresponding score. The test was repeated three times, and the score (0–4) was decided by the walking distance. The average score of three consecutive trials was calculated. Higher scores indicated better test performance. The scores are as follows: 0 point: the mouse cannot grasp or sit on the wooden pole and falls immediately; 1 point: the mouse can grasp or sit on the wooden pole and cannot move, but can remain on the pole for 1 minute; 2 points: the mouse can maintain its balance on the wooden pole, carry out small activities, and remain on the pole for 1 minute; 3 points: the mouse can walk from one end of the pole to the other end, but foot faults occur; and 4 points: the mouse can freely walk from one end of the wooden pole to the other [27].

#### 2.4.2. Basso Mouse Scale (BMS)

This open-field locomotor scoring system ranges from 0 (no ankle movement) to 9 (frequent or consistent plantar stepping that is mostly coordinated, parallel paws during the initial contact and lifting of the paws, normal trunk stability, and a constantly raised tail) [28, 29].

All the behavioral tests were randomly assigned, and the investigator was blinded to the tests.

### 2.5. Electrophysiological Assessment

In each recording session, the mice were anesthetized with halothane (70% N_2_O and 30% O_2_; 4% induction, 2% maintenance). Motor-evoked potentials (MEPs) were elicited with a pair of needle monopolar electrodes placed over the intact scalp and implanted into the skull above the primary motor cortex. The cathode was placed at the midpoint of an imaginary line connecting the two ears, and the anode was placed at the base of the nose. A needle electrode was inserted into the contralateral gastrocnemius muscle to record MEPs. Electrical stimulation was applied with a stimulator to excite the brain (Keypoint, Medtronic, USA). A single pulse of stimulation (7.8 mA, 0.1 ms, 1 Hz) was delivered via a single electrode (DSN1620, Medtronic, USA). A single pulse of stimulation (100 *μ*s, 350 V) was delivered via a modified E5-9S ear electrode (Electro-Cap Inc., Eaton, OH, China). The electrical stimulation was repeated five times in each mouse with an interval of 15 seconds. An activity that was two standard deviations above the baseline activity in response to transcranial stimulation was regarded as the MEPs. The MEP latency was recorded for analysis.

Clampfit software was used to analyze MEP data. Events that occurred within 4–8 ms after TMS and had the peak amplitude that was two standard deviations above the baseline activity were regarded as MEPs.

### 2.6. Immunoblot Analysis

Cultured cells or tissues were solubilized in sample buffer, and the protein concentration of each sample was determined using a Beyotime protein assay kit with bovine serum albumin as the standard. Immunoblot analysis (30 *μ*g of protein per lane) was conducted using a 4–10% SDS gradient polyacrylamide gel followed by a standard blotting procedure. Primary antibodies that selectively recognize goat anti-MBP (1 : 250; Santa Cruz; Cat: sc-13914, USA) and actin (1 : 2000, Santa Cruz, USA) were used. Images of the blots were analyzed using ImageJ software (NIH, USA).

### 2.7. Cell Cultures and In Vitro Study

#### 2.7.1. Cell Cultures

OLI-neu, an immortalized oligodendrocyte cell line, was kindly gifted by Professor Lan Xiao (Department of Histology and Embryology, Chongqing Key Laboratory of Neurobiology, Third Military Medical University) and cultured in DMEM (Gibco, USA) with 10% fetal calf serum (FCS; Gibco, USA), N2 supplement (Gibco, USA), and insulin (Sigma, USA). For the cell death experiment and mitochondrial membrane potential, the OLI-neu cells were exposed to 250 *μ*M FeCl_2_ for 48 hours with or without 400 nM MitoQ and 1 mM NAC (N-acetyl-L-cysteine; Sigma, USA). For ATP and mitochondrial ROS assays, OLI-neu cells from each group were treated as described above for 24 hours.

#### 2.7.2. Mitochondrial Membrane Potential and Cell Death Assay

The mitochondrial membrane potential (*∆*Ψm) was measured using TMRM (Life Technology, USA) following the manufacturer's instructions. The OLI-neu cells were plated at a concentration of 1 × 10^5^ cells/ml. The cells were then stained with TMRM at a final concentration of 25 nM in the culture medium at room temperature. TMRM staining was analyzed using the Auto Live-Cell Imaging Station (Invitrogen™, USA), with excitation and emission wavelengths of 535 nm and 610/620 nm, respectively [7]. Cell death analysis was performed using a propidium iodide (PI; Thermo, USA) detection kit (R37108, Thermo, USA); at the end of the experiment, the medium was removed, and the cells were washed with PBS and stained with 500 nM PI for 20 min in a humidified atmosphere of 5% CO_2_ at 37°C. After washing with PBS, the cell sampling was performed using flow cytometry (Beckman MoFlo XDP, USA).

#### 2.7.3. Mitochondrial ROS and ATP Content Detection

Mitochondrial ROS were measured by MitoSOX Red (Molecular Probes, Eugene, OR, USA), which is a fluorogenic indicator of superoxide generated specifically by the mitochondria. At the end of the experiment, the medium was removed, and the cells were washed with PBS and stained with 5 *μ*M MitoSOX Red for 10 min in a humidified atmosphere of 5% CO_2_ at 37°C. After washing with PBS, the cell sampling was performed using confocal microscopy (Zeiss LSM 780, Germany) or flow cytometry (Beckman MoFlo XDP, USA) and analyzed by FlowJo. For in vivo Mito-ROS (mitochondrial ROS) analysis, perihematomal tissues were homogenized in mitochondria isolation buffer reagent A (Mitochondria Isolation Kit, 89874, Thermo, USA) and centrifuged at 850*g* for 5 min at 4°C. The pellet was discarded, and the supernatant was centrifuged a second time at 13,500*g* for 10 min. The pellet was resuspended in isolation buffer reagent C, and the mixture was centrifuged again at 13,500*g* for 10 min. This step was repeated once, and the final pellet was resuspended in isolation buffer without EDTA, then washed with mitochondrial solution and stained with 5 *μ*M MitoSOX Red for 10 min in a humidified atmosphere of 5% CO_2_ at 37°C. After washing with PBS, the cell sampling was performed using confocal microscopy.

OLI-neu cells were lysed in RIPA buffer and homogenized by passing through a 25-gauge syringe needle multiple times. The samples were then centrifuged at 10000 rcf (*g*) for 10 min at 4°C, and then ATP levels in the supernatants were measured using a bioluminescence detection kit (Beyotime, China). In brief, 30 *μ*l (cells) of supernatant was transferred into one well of a black 96-well plate, and 150 *μ*l reaction buffer was added to each well. The luminescence was determined as relative luminescent units using a Varioskan Flash (Thermo, USA). Experiments were performed with two replicates of each sample. The determined protein concentrations of each sample were used to normalize the ATP level.

### 2.8. Statistical Analysis

The values are presented as the mean ± SEM, and SPSS 19 (SPSS Inc., Chicago, USA) was used for statistical analysis. If the data were not normally distributed even with log transformation, the Kruskal-Wallis test followed by Dunn's post hoc test was used for statistics, and the median and interquartile range are used to express this data. The Mann–Whitney *U* test was used to compare behavioral and activity scores among the groups. Other data were analyzed by one-way ANOVA followed by the Scheffé *F* test for post hoc analysis or by Student's *t* test. *P* < 0.05 was considered statistically significant.

## 3. Results

### 3.1. MitoQ Attenuated Neurological Deficits after ICH

**Figure 1:**
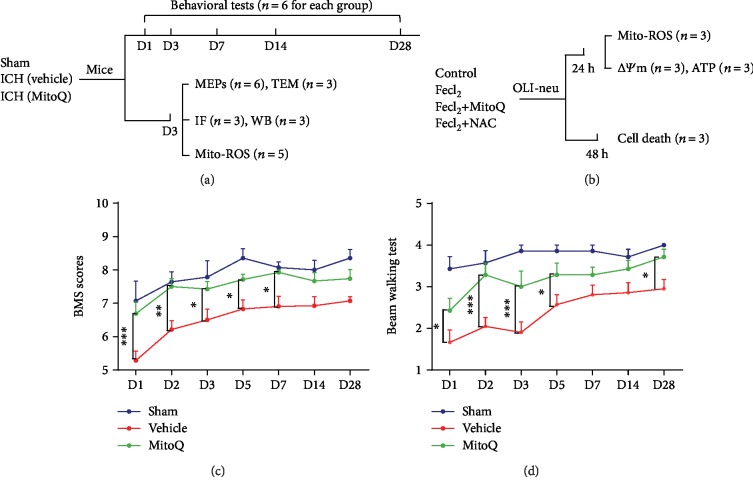
Mitoquinone (MitoQ) attenuated neurological deficits after ICH. (a) In vivo experimental design. IF: immunofluorescence; WB: western blotting. The “*n*” means the number of mice in each group. (b) In vitro experimental design. (c) Basso Mouse scale. (d) Beam walking test. The neurological score data is expressed as the median and the interquartile range and were analyzed using the Kruskal–Wallis test followed by Dunn's post hoc test. ^∗^*P* < 0.05; ^∗∗^*P* < 0.01; ^∗∗∗^*P* < 0.001.

The Basso Mouse Scale (BMS) and the beam walking test indicated neurological function impairments in the ICH+vehicle group compared to the sham group (Figures 1(a) and 1(b)). The MitoQ treatment group exhibited improved neurological scores compared to those of the ICH+vehicle group (BMS, *P* < 0.05 on days 1, 2, 3, 5, and 7; beam walking, *P* < 0.05 on days 1, 2, 3, 5, and 28; Figures 1(c) and 1(d)).

### 3.2. MitoQ Alleviated MEP Latency Prolongation and White Matter Damage after ICH

**Figure 2:**
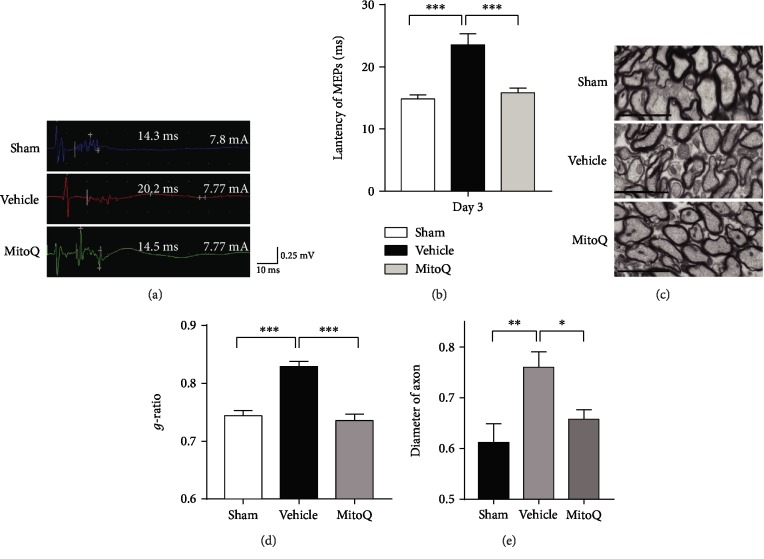
Mitoquinone (MitoQ) attenuated latency prolongation and white matter injury after ICH. (a) Motor-evoked potential latency. The scale bar is shown adjacent to the image. (b) The results of the latency of MEPs were analyzed; *n* = 6 per group. (c) Transmission electron microscopy showing axons and myelin sheaths around the hematoma on the third day after ICH; scale bars: 2 nm (inset column). (d) The *g*-ratios of the different groups; *n* = 60, 3 mice. (e) The diameters of the axons; *n* = 60, 3 mice. The values represent the mean ± SEM when using ANOVA followed by Tukey's post hoc test. ^∗^*P* < 0.05; ^∗∗^*P* < 0.01; ^∗∗∗^*P* < 0.001.

Our results and other research groups found that the neurological behavior and electrophysiological conductivity of mice after ICH was significant impaired after three days. In addition, the pathological results of previous studies also showed that the inflammatory reaction and white matter damage around the hematoma were the most serious on the third day [30, 31]. So we chose the third day after bleeding as the main time to study the physiological changes. The motor-evoked potential (MEP) latency, which indicated the transduction of cortical spinal tracts, was significantly prolonged on the third day after ICH (23.56 ± 1.74 ms in the vehicle group versus 14.84 ± 0.67 ms in the sham group, *P* < 0.001; Figures 2(a) and 2(b)), and MitoQ treatment reduced the MEP latency prolongation (15.84 ± 0.78 ms in the MitoQ group versus the vehicle group, *P* < 0.001; Figures 2(a) and 2(b)). The *g*-ratio of the nerve conduction tracts was calculated in the internal capsule around the hematoma and an increased *g*-ratio indicated thinning of the myelin sheath (sham, 0.61 ± 0.037; vehicle, 0.76 ± 0.031; MitoQ, 0.66 ± 0.019; Figures 2(c) and 2(d)). The diameter of the axons is listed in each group, and an increase in the diameter of axon indicates the axon edema (sham, 0.74 ± 0.009; vehicle, 0.82 ± 0.008; MitoQ, 0.73 ± 0.010; Figure 2(e)). The results showed that the *g*-ratio of the tracts and the diameter of the axons were significantly increased in the ICH+vehicle group compared with the sham group (diameter of the axons, *P* < 0.01; *g*-ratio, *P* < 0.001; Figures 2(c)–2(e)). Compared with vehicle treatment, MitoQ treatment resulted in a significant increase in the *g*-ratio of the tracts and the diameter of the axons when compared with the ICH+vehicle group (diameter of the axons, *P* < 0.05; *g*-ratio, *P* < 0.001; Figures 2(c)–2(e)).

### 3.3. MitoQ Reduced Demyelination after ICH

**Figure 3:**
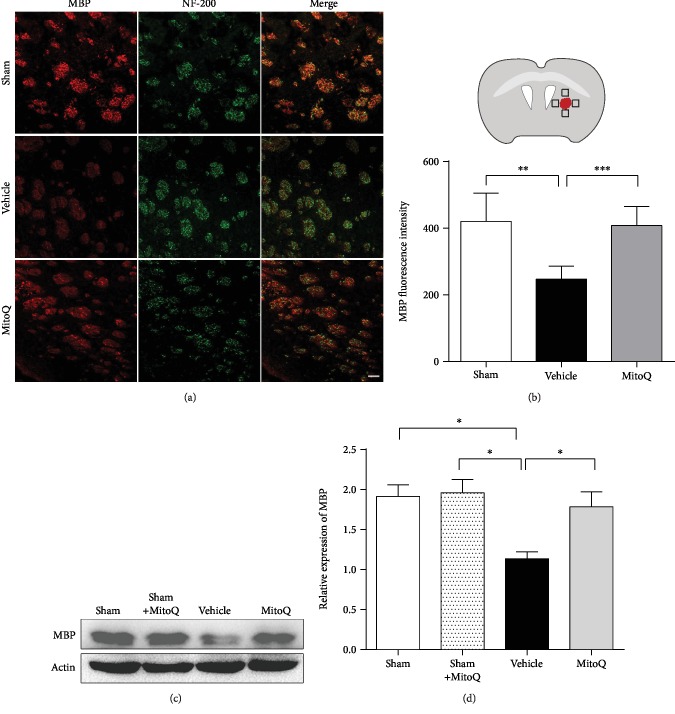
Mitoquinone (MitoQ) reduced myelin degradation after ICH. (a) Representative images of double immunofluorescence staining for MBP and NF-200 on the third day after ICH. Scale bars: 50 *μ*m (inset column). (b) The quantification of MBP staining (right); *n* = 3 mice and 6 pictures. (c) Representative western blot images. Quantitative analyses of (d) MBP expression; *n* = 5 per group. The values represent the mean ± SEM; ^∗^*P* < 0.05; ^∗∗^*P* < 0.01; ^∗∗∗^*P* < 0.001. The black box in panel (b) shows the region of interest in the brain.

The intensity of myelin basic protein (MBP) staining in the vehicle group was decreased on day 3 compared with that in the sham group (*P* < 0.01) (Figures 3(a) and 3(b)), which indicated the degradation of myelin. Western blotting confirmed a significant decrease in the expression of MBP in the ICH+vehicle group compared with the sham group (*P* < 0.05; Figures 3(c) and 3(d)). And compared to vehicle treatment, the administration of MitoQ after ICH resulted in a significant increase in MBP expression compared with the ICH+vehicle group (*P* < 0.05; Figures 3(c) and 3(d)).

### 3.4. MitoQ Decreased the Loss of Oligodendrocyte Precursor Cells and Oligodendrocytes after ICH

**Figure 4:**
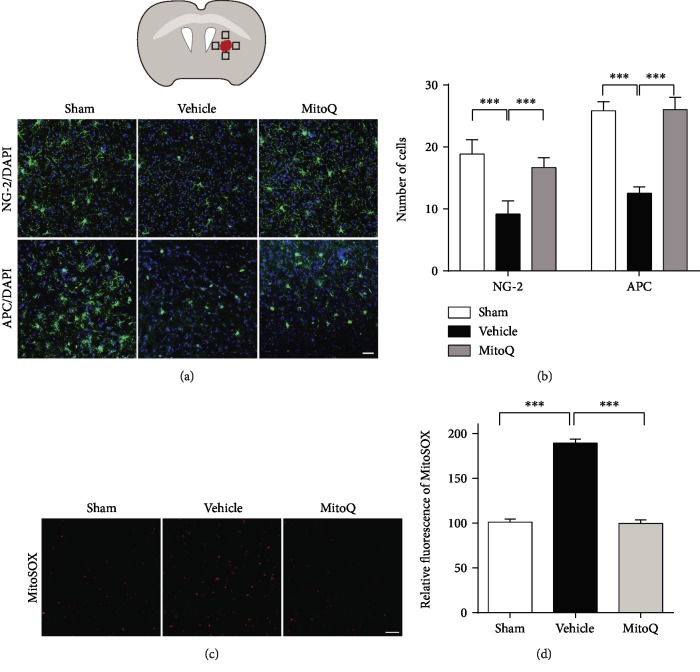
MitoQ decreased the loss of oligodendrocyte precursor cells (OPCs) and oligodendrocytes 3 days after ICH. (a) Images showing NG-2 or APC immunostaining (green) with DAPI (blue) in the internal capsule of the mice after ICH. (b) The results of the quantitative analysis of the number of APC-positive and NG-2 positive cells in bin areas of the internal capsule; *n* = 6 sections, 3 mice per group. (c) The mitochondrial ROS around hematoma were detected by MitoSOX Red 3 days after ICH. (d) The intensity of MitoSOX Red was analyzed show in histogram; *n* = 5 mice. The values represent the mean ± SEM. Scale bar: (a) 50 *μ*m, (c) 20 *μ*m. ^∗^*P* < 0.05; ^∗∗^*P* < 0.01; ^∗∗∗^*P* < 0.001. The black box in panel (a) shows the region of interest in the brain.

Three days after ICH, the mice were euthanized, and their brains were processed for the analysis of cell survival in the regions around the hematoma of the internal capsule. The brain sections were stained with DAPI to label all of the cells and with NG-2 and APC antibodies to label oligodendrocyte precursor cells (OPCs) and oligodendrocytes (OLs), respectively (Figure 4(a)). The results of the cell counts revealed that significantly more OLs and OPCs loss in the ICH+vehicle group mice compared to the sham group mice (OPCs, *P* < 0.001; OLs, *P* < 0.001; Figures 4(a) and 4(b)). MitoQ treatment attenuated the loss of both cell types (OPCs, *P* < 0.05; OLs, *P* < 0.05; Figures 4(a) and 4(b)). The level of perihematomal mitochondrial ROS increased 3 days after ICH injury in mice. MitoQ, a selective mitochondrial ROS scavenger, significantly reduced the level of mitochondrial ROS (Figures 4(c) and 4(d)).

### 3.5. MitoQ Blocked Iron Overload-Induced Loss of Mitochondrial Membrane Potential and Death of OLI-Neu Cells

Cell death caused by oxidative stress is one of the mechanisms of secondary injury after ICH, and mitochondrial dysfunction is the important target of cell death caused by oxidative stress. To verify the protective effect of selective mitochondria ROS scavengers on oxidative stress after ICH, iron, which is released from hematoma after ICH, was used to simulate oxidative stress injury. TMRM was used to detect mitochondrial membrane potential and NAC as a nonselective ROS scavenger as control.

**Figure 5:**
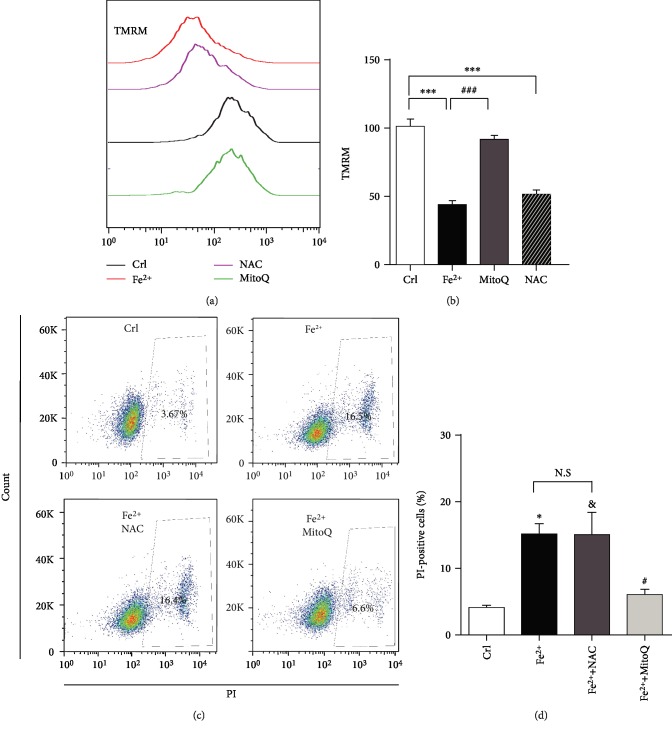
MitoQ protected OLI-neu cells from mitochondrial membrane potential decrease and cell death caused by FeCl_2_. (a) After 24 hours of treatment with FeCl_2_ (250 *μ*M) and cotreatment with or without NAC (1 mM) or MitoQ (200 *μ*M), TMRM fluorescence was detected in OLI-neu cells by flow cytometry. (b) Quantitative analyses of TMRM fluorescence; *n* = 6. Scale bars: 20 *μ*m. The data are expressed as means and SEM and were analyzed using ANOVA followed by Tukey's post hoc test. (c) OLI-neu cells were treated with FeCl_2_ and with or without NAC (1 mM) or MitoQ (200 *μ*M). Cell death was detected by propodium iodide staining and flow cytometry after 48 hours. (d) Quantitative analyses of PI-positive cells, *n* = 3. The data are expressed as mean and SEM and were analyzed using 2-way ANOVA followed by Tukey's multiple comparisons test. ^∗^*P* < 0.05; ^∗∗∗^*P* < 0.001 represents Fe^2+^ versus control; ^#^*P* < 0.05, ^###^*P* < 0.001 represents Fe^2+^ versus Fe^2+^+MitoQ; ^&^*P* < 0.05, ^&&&^*P* < 0.001 represents Fe^2+^+NAC versus control.

The real-time detection of living cells showed that MitoQ, but not NAC, could reduce the mitochondrial membrane potential decline and cell death induced by 250 *μ*m FeCl_2_ in OLI-neu cells (cell death rate: 3.67 ± 0.34% in the control group versus 15.13 ± 1.57% in the FeCl_2_ group, *P* < 0.05; 6.07 ± 0.80% in the FeCl_2_+MitoQ group versus 15.03 ± 3.38% in the FeCl_2_+NAC group, *P* < 0.05; Figures 5(a)–5(d)).

### 3.6. MitoQ Inhibited Mitochondrial ROS and ATP Deletion in OLI-Neu Cells

**Figure 6:**
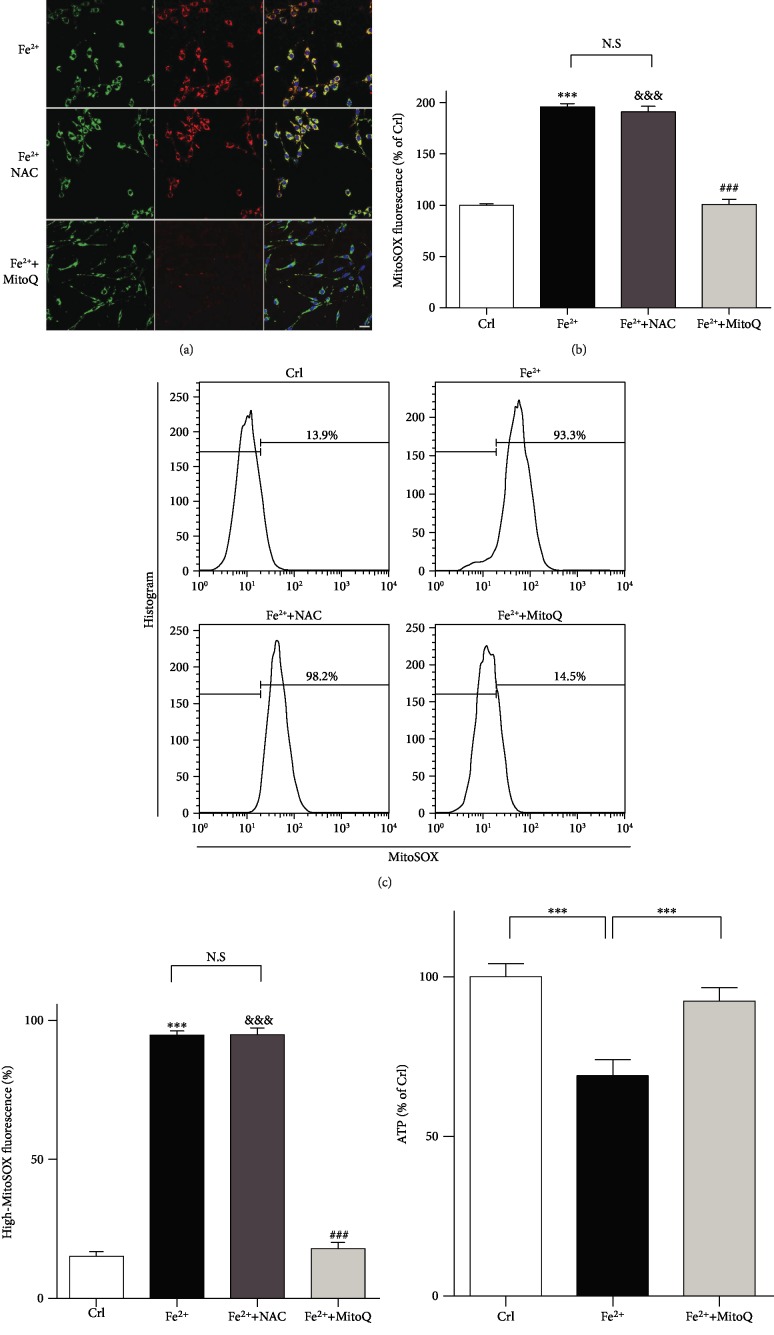
MitoQ decreased the Fecl_2_ insult-induced accumulation of mitochondrial ROS and ATP deletion in OLI-neu cells. The level of mitochondrial ROS accumulation (red) in the OLI-neu cells was examined using MitoSox Red staining 24 hours after FeCl_2_ (250 *μ*M) treatment and treatment with or without NAC (1 mM) or MitoQ (200 *μ*M). The staining was analyzed by microscopy (a) and flow cytometry (c). The data were analyzed in (b) and (d). (e) ATP content was analyzed using in OLI-neu cells after 24 hours of FeCl_2_ treatment and treatment with or without NAC (1 mM) or MitoQ (200 *μ*M). Scale bars in (a) and (c): 20 *μ*m. The data are expressed as mean and SEM and were analyzed using 2-way ANOVA. ^∗∗∗^*P* < 0.001 represents Fe^2+^ versus control; ^###^*P* < 0.001 represents Fe^2+^ versus Fe^2+^+MitoQ; ^&^*P* < 0.05, ^&&&^*P* < 0.001 represents Fe^2+^+NAC versus control.

In order to investigate the protective effect of selective mitochondrial antioxidants, mitochondrial ROS in OLI-Neu cells was detected with MitoSOX after 24 hours of ferrous ion treatment. Results showed that the fluorescence intensity of MitoSOX was significantly enhanced which indicated the increasing of mitochondrial ROS in the OLI-neu cells after treatment with 250 *μ*M Fecl_2_. MitoQ, rather than NAC, can reduce the content of mitochondrial ROS of OLI-neu cells under high ferrous environment (Figures 6(a)–6(d)). The ATP content of OLI-neu cells decreased after treatment with 250 *μ*M Fe^2+^. MitoQ, rather than NAC, increased the content of ATP in OLI-neu cells under high ferrous environment (Figure 6(e)).

## 4. Discussion

The human brain accounts for 20% of the total oxygen consumption of the human body and it has strong oxidative respiration. Brain tissue is rich in polyunsaturated fatty acids, which are sensitive to oxidation, making it most vulnerable to ROS damage [32, 33]. Such as in Parkinson's disease (PD) and amyotrophic lateral sclerosis, the lipid peroxidation marker 4-hydroxysterol (4-HNE) and carbonylated proteins increased significantly in the patient's brain lesion area [34–36]. Therefore, antioxidant therapy is indispensable in the treatment of central nervous system diseases. But so far, there is no clinical evidence to show the intervention in this process can effectively reduce damage [37].

In the progress of antioxidant research, the first thing to consider is the different damage mechanism of specific ROS in different diseases. For example, diabetic nephropathy is characterized by hyperactive NADPH oxidase. Therefore, GKT137831 is a promising specific NADPH oxidase inhibitor, which is currently in phase II clinical trials of diabetic nephropathy [38]. Second is the proposed intervention for selective subcellular localization. Mitochondria are ROS-producing and primary oxidative stress-damaging organelles [17, 39]. Clinical trial reported that CoQ10-targeted mitochondrial ROS therapy to reduce major adverse cardiovascular events, hospitalization rates, and mortality. [40]. Iron overload after ICH was observed in patients as well as in animal models and was associated with excessive ROS production around the hematoma [41, 42]. Although clinical studies of the nonselective reactive oxygen scavenger edaravone have failed [11, 12], interventions that selectively target mitochondrial ROS or specific oxidants are still promising treatments for ICH.

^•^OH is the most oxidative damaging molecule, a key molecule of mitochondria and cell damage caused by iron overload after ICH [43]. It is known that mitoquinone (MitoQ) inhibits the final step of lipid peroxidation by blocking •OH attack and continuously circulates through the return of the mitochondrial respiratory chain complex II to the active panthenol form [19]. MitoQ is a mitochondria-targeting antioxidant that acts as a lipophilic conjugated compound that readily accumulates high concentrations in the mitochondria, where the ubiquinone is reduced to its active antioxidant ubiquinol form [20, 44]. The biological properties of MitoQ not only allow it to cross the mitochondrial membrane but also better access to brain tissue through the blood-brain barrier, which is used in central nervous system diseases [45]. Interventions that selectively target mitochondrial ROS and specific hydroxyl radical damage may make MitoQ suitable for the treatment of oxidative stress damage in intracerebral haemorrhage. Our results showed that the mitochondrial antioxidant MitoQ, but not the nonselective antioxidant NAC, can reduce mitochondrial ROS levels and attenuate the mitochondrial membrane potential loss and cell death induced by ferrous overload in OLI-neu cells. In addition, in vivo data proved that MitoQ can inhibit loss of oligodendrocyte precursor cells and oligodendrocytes after ICH.

The basal ganglia haemorrhage accounts for more than 80% in all the intracerebral parenchymal haemorrhage [46]. The basal ganglia is full with corticospinal tracts, so WMI-induced motor deficit is a serious complication [2, 47]. Demyelination severely impairs white matter conduction and is considered to underline the long-term neurological deficits in central nervous system diseases such as intracerebral hemorrhage (ICH) and multiple sclerosis [30, 48]. The mechanism of WMI after ICH has not been elucidated and there is no specific intervention. The relationship between WMI and mitochondrial ROS after ICH is unclear [49]. It was reported that myelin degradation may be involved in WMI after ICH [50]. Our results showed that severe myelin degradation, axon swelling, and motor-evoked potential (MEP) latency prolongation occurred on the third day after ICH, which correspond to behavioral impairment. And the present data illustrated that MitoQ significantly reduced the myelin loss and oligodendrocyte loss, and ultimately promoted the integrity of myelin-axon in the white matter after intracerebral hemorrhage in the basal ganglia, thereby improving conduction velocity of the corticospinal tract and neurological function in mice. To our knowledge, this study is the first to reveal the protective effect and the underlining of MitoQ on white matter injury after ICH.

**Figure 7:**
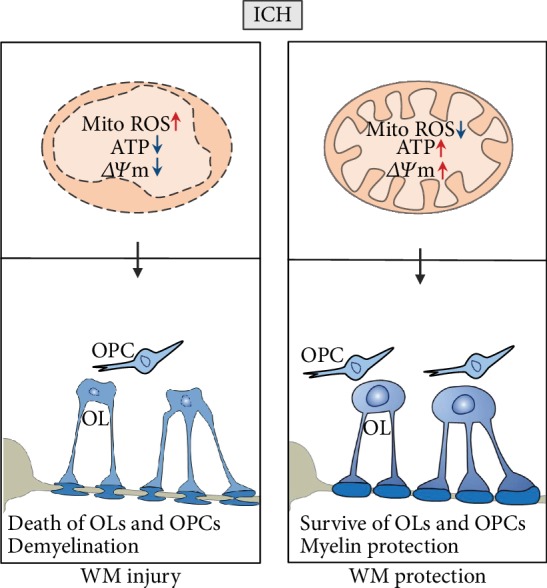
Intracerebral haemorrhage was accompanied demyelination and loss of oligodendrocytes (OLs) and oligodendrocyte precursor cells (OPCs), which resulted in white matter injury. MitoQ treatment reduced OL and OPC death by blocking iron overload-induced mitochondrial damage, which include the increase of mitochondrial ROS and decrease of ATP and mitochondrial membrane potential (*∆*Ψm). Finally, rescuing white matter injury improved the outcomes after intracerebral haemorrhage.

In summary, we demonstrated that selective mitochondrial ROS scavenger MitoQ can attenuate white matter injury and improve neurological impairment after ICH. This may due to MitoQ administration-reduced oligodendrocyte death and demyelination after ICH through the inhibition of mitochondrial injury after ICH. Therefore, MitoQ can be used as a therapeutic agent for neuroprotection after ICH (Figure 7).

## Data Availability

We can provide the data if needed.
